# Improvement of Certolizumab Fab′ properties by PASylation technology

**DOI:** 10.1038/s41598-020-74549-0

**Published:** 2020-10-28

**Authors:** Somayeh Mazaheri, Yeganeh Talebkhan, Fereidoun Mahboudi, Leila Nematollahi, Reza Ahangari Cohan, Esmat Mirabzadeh Ardakani, Elham Bayat, Masoumeh Sabzalinejad, Soroush Sardari, Fatemeh Torkashvand

**Affiliations:** 1 Biotechnology Research Center, Pasteur Institute of Iran, Tehran, Iran; 2 Department of Nanobiotechnology, Advanced Technology Group, Pasteur Institute of Iran, Tehran, Iran; 3 Department of Molecular Medicine, Pasteur Institute of Iran, Tehran, Iran

**Keywords:** Biotechnology, Biologics, Antibody therapy

## Abstract

Certolizumab pegol is a Fab′ antibody fragment for treatment of rheumatoid arthritis and Crohn’s disease which is conjugated to a 40 kDa PEG molecule in order to increase the protein half-life. PEGylation may have disadvantages including immunogenicity, hypersensitivity, vacuolation, decreased binding affinity and biological activity of the protein. To overcome these problems, PASylation has been developed as a new approach. The nucleotide sequence encoding 400 amino acid PAS residues was genetically fused to the corresponding nucleotide sequences of both chains of certolizumab. Then, the bioactivity as well as physicochemical and pharmacokinetic properties of the recombinant PASylated expressed protein was assayed. Circular dichroism spectroscopy demonstrated that the random coil structure of PAS sequences did not change the secondary structure of the PASylated Fab′ molecule. It was observed that PASylation influenced the properties of the Fab′ molecule by which the hydrodynamic radius and neutralization activity were increased. Also, the antigen binding and binding kinetic parameters improved in comparison to the PEGylated Fab′ antibody. Pharmacokinetic studies also showed prolonged terminal half-life and improved pharmacokinetic parameters in PASylated recombinant protein in comparison to the PEGylated and Fab′ control molecules. The results reconfirmed the efficiency of PASylation approach as a potential alternative method in increasing the half-life of pharmaceutical proteins.

## Introduction

Malfunctioning of the immune system is the primary cause of several inflammatory diseases including rheumatoid arthritis (RA), Crohn’s disease (CD), and ankylosing spondylitis (AS)^1,2^. Rheumatoid arthritis has been characterized by inflammatory reactions within peripheral joints which can damage the cartilage thus leading to disability and decreased physical function^3,4^. Crohn’s disease is a progressive and destructive disease characterized by induced inflammation in the gastrointestinal tract from the mouth to the anus^5^. The main reason in development of these diseases is the incorrect expression of inflammatory cytokines, especially the tumor necrosis factor-α (TNF-α)^1,2^. TNF-α, an important pro-inflammatory cytokine generated by macrophages and lymphocytes, has been suggested as a prime target for the treatment of the mentioned inflammatory diseases^6,7^. There are five FDA approved anti-TNF-α agents including infliximab, adalimumab, golimumab, certolizumab pegol, and etanercept^2,8^.

Among all the mentioned anti-TNF-α agents, only Certolizumab pegol (Cimzia) is produced in *E. coli* while others are produced in mammalian cells. Production of monoclonal antibodies in eukaryotic cells requires laborious processes and high cost because of the molecule complexity. In general, protein expression in *E. coli*, as a prokaryotic cell, has many advantages over the eukaryotic cells including lower cost of the process, easy transformation and fermentation, simple culture media, rapid growth, and higher yield of the product^9^. Certolizumab pegol is a recombinant, humanized, PEGylated Fab′ antibody fragment. Unlike other anti TNF-α molecules, certolizumab pegol does not have the Fc antibody region nor does it induce Fc-mediated effects in complement-dependent cytotoxicity (CDC) and antibody-dependent cell-mediated cytotoxicity (ADCC). On the other hand, cellular apoptosis has been documented through unknown mechanisms^8,10^. Meanwhile, given the small size of this molecule (~ 50 kDa), a short half-life has been assumed where rapid removal from kidney and blood circulation is inevitable. To overcome this problem, the C-terminal part of the Fab′ fragment has been covalently fused to a single 40 kDa PEG molecule. PEG is a biocompatible polymer used to enhance the pharmacokinetics of small therapeutic proteins^11,12^. The PEG polymer, as a conjugate, confers some advantages to proteins such as increasing half-life, hydrodynamic volume and solubility, while PEGylation harbors disadvantages including immunogenicity, hypersensitivity, vacuolation, decreased binding affinity and biological activity due to the shielding effect and increased viscosity of the sample (reviewed by Zhang et al*.*)^13^. Note that the conjugation procedure usually occurs after purification of the protein through chemical reactions which makes the process more expensive and complex to remove unwanted products^12–15^. Over the last two decades, biological alternative strategies have been developed to extend the protein half-life including XTENylation, ELPylation, HAPylation, dextran conjugation, transferrin fusion, glycosylation, polysialylation, HESylation, HEPylation, HAylation, PASylation or fusion of the desired protein to Gelatin-like protein (GLK), Albumin binding domain, or antibody Fc fragment^16^.

PASylation is a new approach to boost the plasma half-life of small proteins where repeated sequences of three hydrophilic amino acids including proline, alanine and serine will form random coil secondary structures. PASylation has demonstrated all features of PEGylation approach and to a lesser extent its disadvantages. In brief, it does not require any chemical conjugation reaction or further purification steps. PAS sequences are not degraded by serum proteases but can be degraded by kidney proteases. They do not accumulate within tissues and are not immunogenic in the human body due to their nature^17–19^.

The goal of the present study was to determine the physicochemical and biological characteristics of PASylated certolizumab Fab′ protein (200 related amino acids were fused to C-terminal of heavy and light chains) and to assess its in vivo pharmacokinetic parameters in comparison to the control PEGylated (Cimzia) and Fab′ molecules.

## Results

### Cloning of PASylated Fab′ cassette

Restriction digestion and sequencing confirmed that subcloning was done successfully and heavy chain sequence was integrated upstream the light chain sequence in the final pET28a expression vector (data not shown).

### Expression of PASylated Fab′ antibody

**Figure 1:**
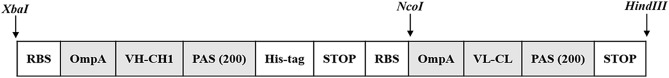
Final gene cassette expressing the PASylated Fab′ fragment in pET28a.

The final plasmid (Fig. 1) was transformed into *BL21 (DE3)* competent cells for periplasmic expression of the PASylated Fab′ protein.

**Figure 2:**
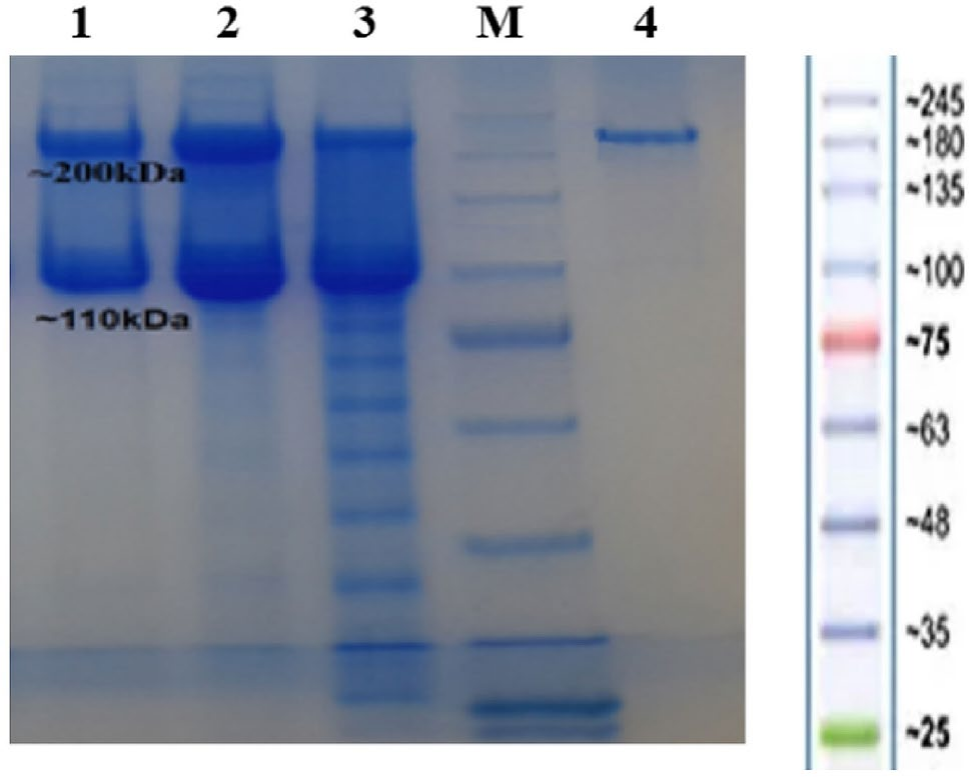
Analysis of protein purification process (non-reducing SDS-PAGE): 1: Eluted protein from one step Ni–NTA chromatography (~ 200 kDa band of PASylated Fab′ and ~ 110 kDa band of PASylated heavy chain); 2: Eluted protein from one step KappaSelect chromatography (~ 200 kDa band of PASylated Fab′ and ~ 110 kDa band of PASylated light chain); 3: Initial sample (IS) originated from the culture medium, M: Protein Mw marker, 4: Eluted ~ 200 kDa PASylated Fab′ from two-step purification procedure (KappaSelect followed by Ni–NTA affinity chromatography).

**Figure 3:**
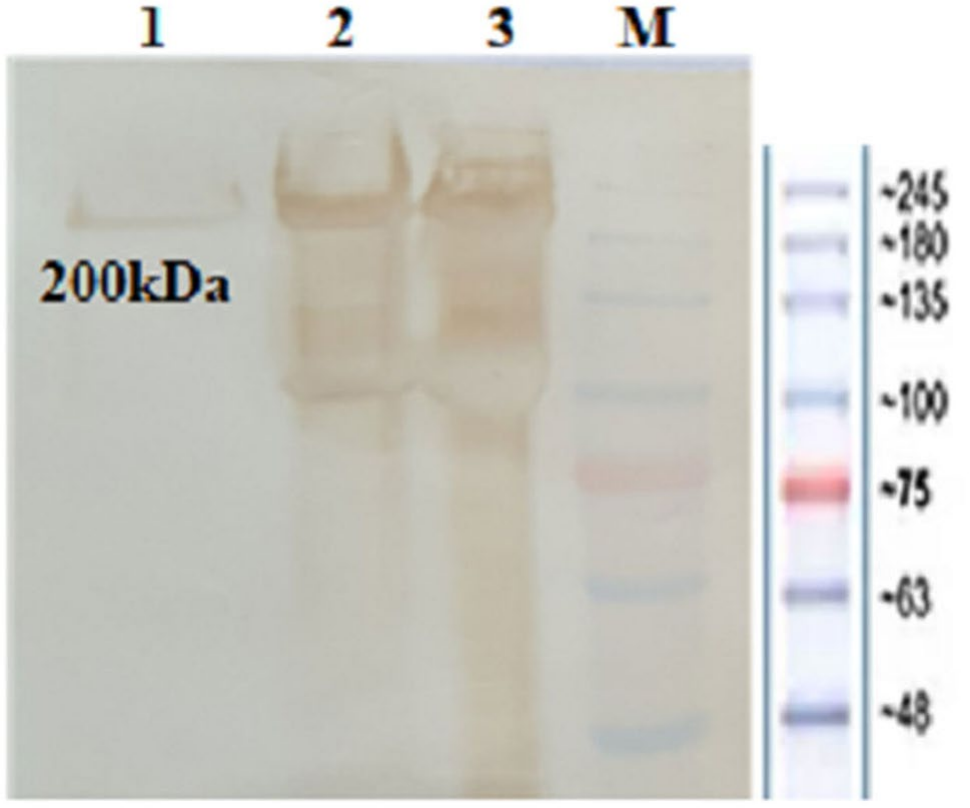
Analysis of protein purification process through Western blotting (non-reducing SDS-PAGE): 1: Eluted ~ 200 kDa PASylated Fab′ from two-step purification procedure (KappaSelect followed by Ni–NTA affinity chromatography); 2, 3: Eluted proteins from one step Ni–NTA chromatography (~ 200 kDa band of PASylated Fab′ and ~ 110 kDa band of PASylated heavy chain); M: Protein Mw marker. The image is cropped to show the one and two-step purification procedures.

The best conditions for periplasmic expression of the PASylated Fab′ molecule were observed within *BL21 (DE3)* strain, TB medium, 0.5 mM IPTG as the inducer, at OD 600 nm of 0.5 (induction time), and 24 h post-induction at 200 rpm (yield of the expressed protein was 70 mg/l). The eluted protein samples obtained from different affinity columns were analyzed on 8% SDS-PAGE. It was observed that the two-step purification procedure (KappaSelect followed by Ni–NTA affinity chromatography) was more efficient in purification of the PASylated Fab′ molecule with the molecular weight of approximately 200 kDa (Fig. 2). Western blotting with anti-His antibody also confirmed the purified 200 kDa PASylated Fab′ molecule (Fig. 3). Interestingly, the amount of mature protein secreted into the culture medium was greater than the periplasmic mature protein content (data not shown).

### Secondary structural analysis

**Figure 4:**
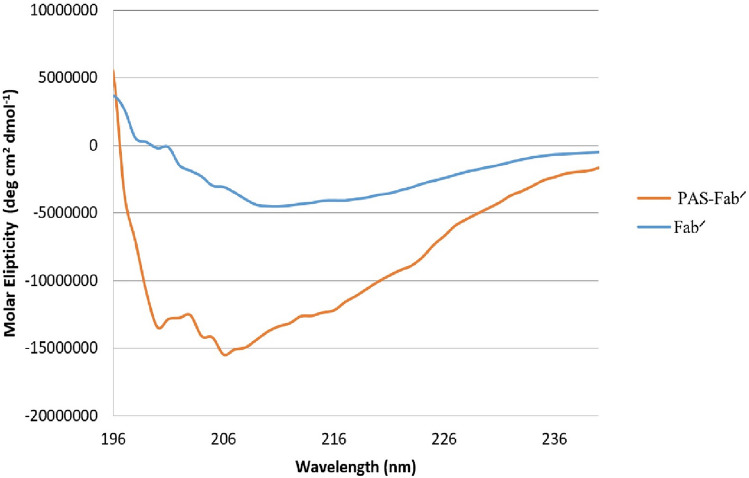
Secondary structure analysis by Far-UV CD spectroscopy: Molar ellipticity calculations demonstrate a negative shift around 200 nm in the curve of PAS-Fab′ compared to its Fab′ antibody.

Circular dichroism (CD) spectroscopy was performed to obtain structural information about the possible conformational effects of PAS sequences (200 residues) added to the end of both light and heavy fragments of the Fab′ in comparison to its corresponding native Fab′ molecule. After measurement of the molar differences, the CD spectrum of the PASylated protein revealed a major negative minimum shift around 200 nm corresponding to an increase in the random coil structure of the protein from 24 to 44% (Fig. 4) in accordance with the previous study performed by Schlapschy et al.^18^. The frequency of random coil formation was higher in PASylated protein which is due to the natural structure of proline, alanine, and serine residues in the PAS fragments.

### PASylated Fab′ TNF-α binding assay

**Figure 5:**
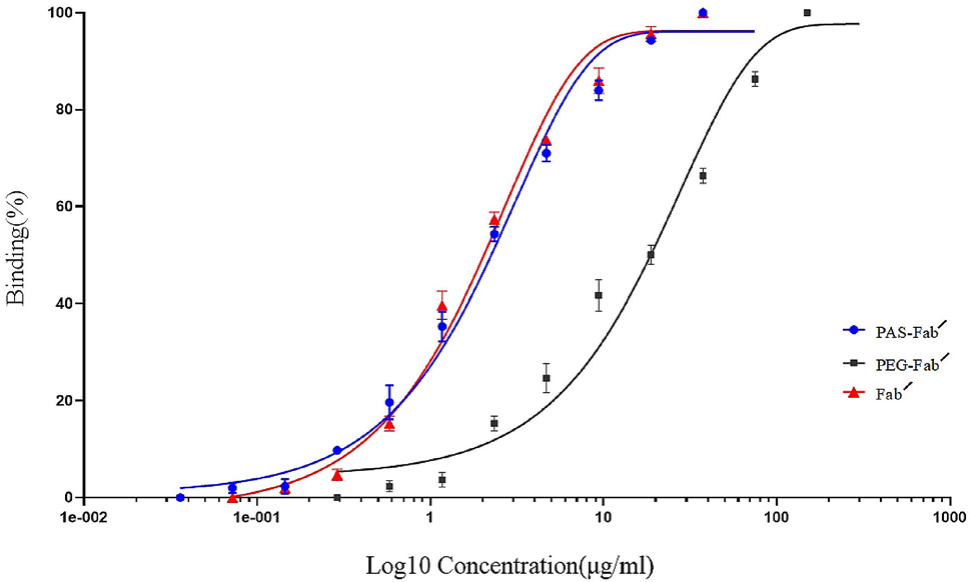
TNF-α ELISA binding activity of PAS-Fab′, PEG-Fab′ and Fab′ antibodies: data are represented as mean ± SD (three replicates).

ELISA test was conducted to assess the affinity of PASylated, PEGylated, and Fab′ antibodies against TNF-α. The results revealed that the addition of two PAS fragments (200 residues to heavy and light fragments) did not inversely affect the antigen-binding activity of the Fab′ molecule. In contrast, the affinity of the PASylated Fab′ antibody showed an approximate tenfold difference to the PEGylated molecule (Fig. 5). The EC50 values of 18.83, 2.07, and 1.99 µg/ml were also calculated for PEGylated, PASylated, and Fab′ antibodies, respectively. These results showed similar affinity towards TNF-α for PASylated and Fab′ molecules which were higher than the affinity of the PEGylated protein.

### Affinity measurement

The affinity of the tested antibodies was measured using Beatty’s protocol as described in the method section. The K_*aff*_ of PASylated, PEGylated, and Fab′ molecules were 1.68 × 10^12^ (M^−1^), 4.43 × 10^11^ (M^−1^), and 1.21 × 10^12^ (M^−1^), respectively.

### Hydrophobicity analysis

**Figure 6:**
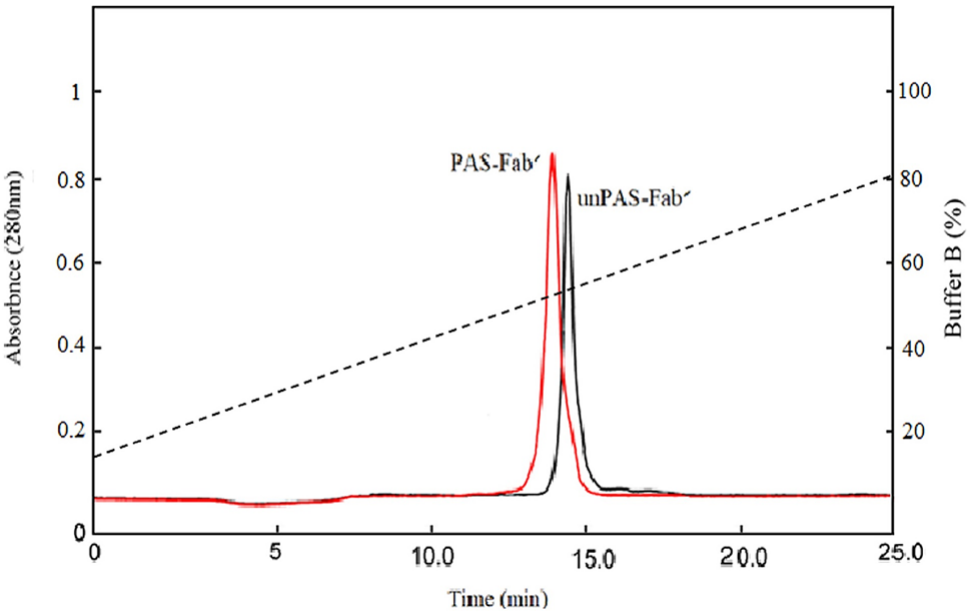
RP-HPLC of PAS-Fab′ molecule in comparison to the corresponding Fab′ antibody: Samples were analyzed with gradient elution (2–80% CAN in 0.065–0.05% TFA) at 280 nm.

The hydrophobicity of the PASylated and Fab′ antibodies was assessed by RP-HPLC. PASylated Fab′ was eluted in 52.1% acetonitrile while its native form (Fab′ molecule) was eluted in 53.8% acetonitrile. The obtained profile showed that the addition of two PAS fragments (400 residues in total) slightly reduced the hydrophobicity of the Fab′ molecule (Fig. 6).

### Hydrodynamic radius distribution and molecular weight determination

**Figure 7:**
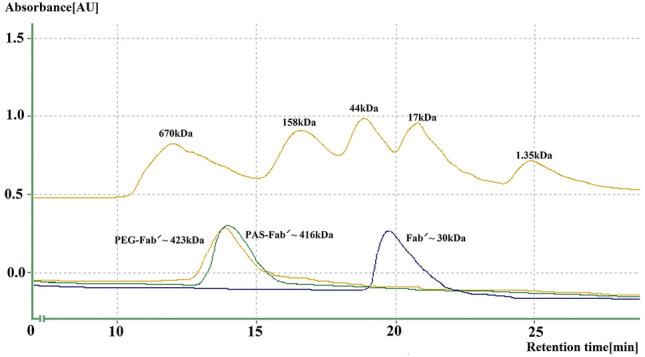
Size exclusion chromatography of PAS-Fab′, PEG-Fab′ and Fab′ antibodies: Mw marker includes the following proteins: Bovine thyroglobulin (670 kDa), bovine c-globulin (158 kDa), chicken ovalbumin (44 kDa), horse myoglobin (17 kDa), vitamin B12 (1.35 kDa).

The hydrodynamic radius of PASylated Fab′ antibody was measured by the analytical SEC method. The actual increased mass of fused PAS fragment (400 amino acids in total) was 34.31 kDa, but the observed apparent mass was 416 kDa representing an approximately 13.86 and 14.1-fold increase in the hydrodynamic radius in comparison to the Fab′ (~ 30 kDa) and PEGylated Fab′ (~ 423 kDa) molecules, due to the random coil structure of the PAS sequence (Fig. 7).

**Figure 8:**
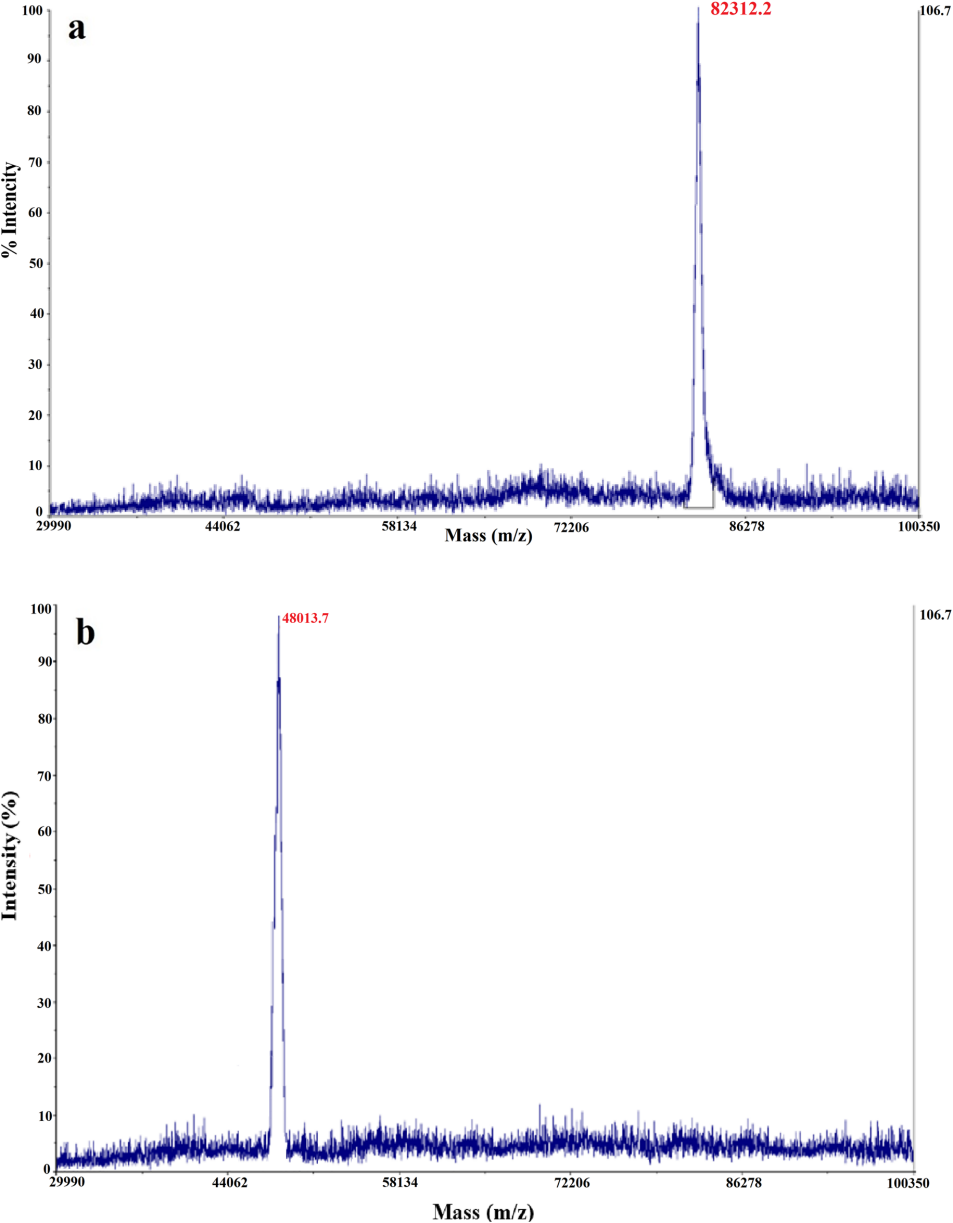
Mass spectrometric analysis by MALDI-TOF/TOF: (**a**) A single peak with a molecular weight of 82,312.2 Da for non-reduced PAS-Fab′ antibody; (**b**) A single peak with a molecular weight of 48,013.7 Da for non-reduced Fab′ antibody.

Mass spectroscopy showed an exact molecular weight of 82,312.2 Da for PASylated Fab′ molecule (Fig. 8) in comparison to the measured 48,013.7 Da Fab′ molecule and the reported 90,000 Da of the PEGylated Fab′. These two tests confirmed the monodisperse composition of the PASylated antibody fragment.

### Neutralization of soluble TNF-α

**Figure 9:**
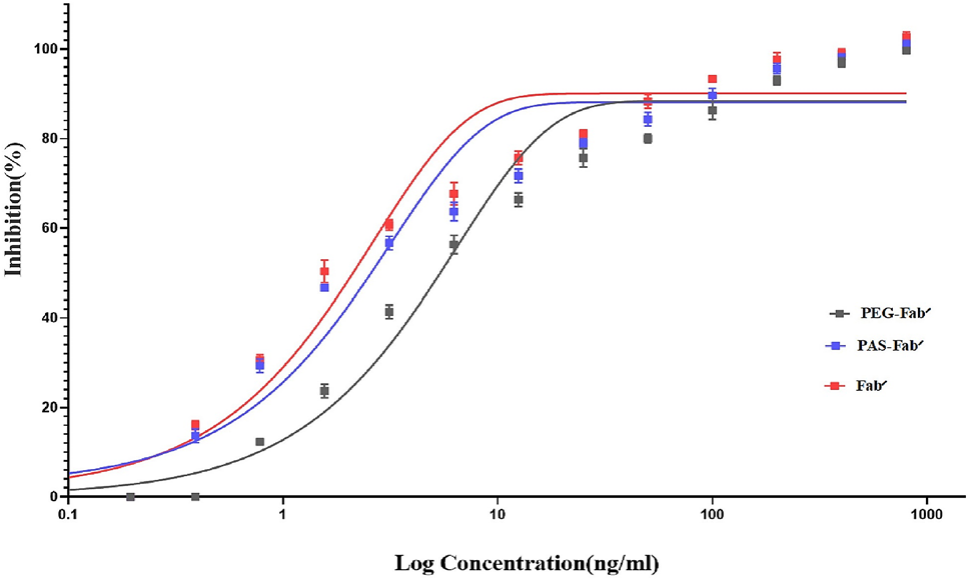
Neutralization of TNF-α-mediated cytotoxicity in L929 cell line.

The binding of TNF-α molecule to its surface receptors has strong cytotoxic effects causing cellular apoptosis. Anti-TNF-α molecules can prevent the cytotoxicity by inhibiting the attachment of soluble TNF-α to the corresponding receptors. In this study, as shown in Fig. 9, a dose-dependent neutralization activity of the tested anti-TNF-α molecules (PEGylated, PASylated and Fab′ antibodies) was observed when 0.5 ng/ml human TNF-α was used. IC50 values of 4.73 2.06, and 1.82 ng/ml were calculated for PEGylated, PASylated, and Fab′ antibodies, respectively. Thus, the activity of PASylated Fab′ antibody was similar to Fab′ and about 2.29 times greater than that of the PEGylated molecule (Fig. 9).

### Thermostability analysis

**Figure 10:**
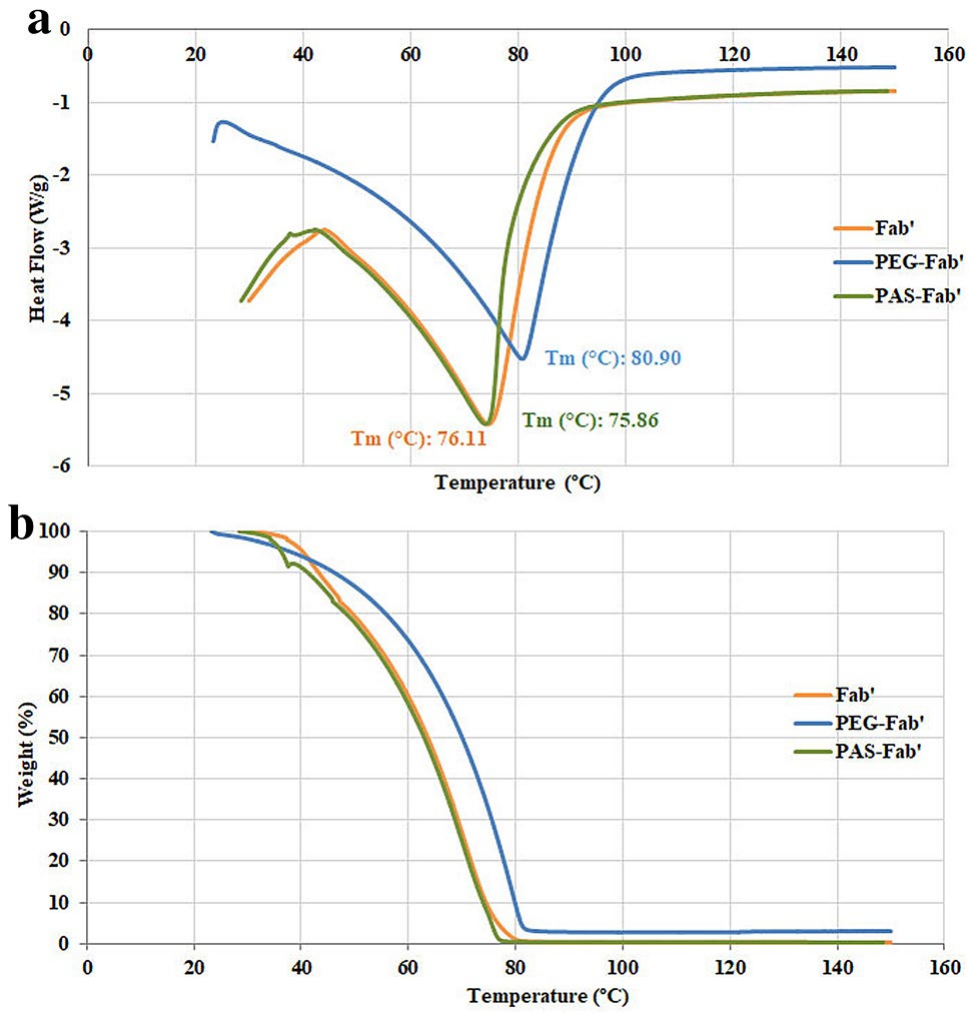
Thermo-analysis of tested PAS-Fab′, PEG-Fab′, and Fab′ antibodies: (**a**) DSC thermograms; (**b**) TG curves: samples were prepared in phosphate buffer (pH7.4) and analysis was performed at a scanning rate of 2 °C/min under nitrogen (inert) atmosphere. The two thermograms differed only to a negligible amount for PAS-Fab′ and Fab′ antibodies while PEG-Fab′ demonstrated a significant shift in comparison to the PAS-Fab′ and Fab′ antibodies regarded to the PEG molecule as a fusion. The melting curves of Fab′, PEG-Fab′, and PAS-Fab′ are highlighted in orange, blue, and green, respectively.

Thermostability is an important parameter for biopharmaceutical proteins which is usually analyzed through dynamic scanning calorimetry (DSC) and thermogravimetry assay (TGA) techniques in which thermodynamic properties of the proteins will be calculated (Tm, ΔT1/2, and ΔHm). The Tm indicates the thermal stability of the proteins which is different from their shelf-life and at temperatures above Tm, irreversible denaturation of the protein happens. In TGA, the mass of the protein will be measured by increasing the temperature at a constant rate. In other words, TGA curves show the initial and maximum temperatures needed for sample degradation. These techniques are indirect tools in evaluation of secondary structural properties of tested proteins^20,21^. The effect of the attached PAS sequence on the thermostability of the Fab′ antibody was investigated using DSC and TGA analysis. Then, the results were compared to the PEGylated form. Denaturation temperature of the PASylated Fab′ was similar to its Fab′ form (in the range of 41–78 °C), while the thermal resistance of the PEGylated Fab′ was greater than that of the other derivatives (from 41 to 87 °C) (Fig. 10a). Fab′ and its PASylated form have similar cooperativity for being unfolded due to the represented similar width of the calorimetric transition at the half peak height which was different from the PEG-Fab′ (Fig. 10a). The ΔHm of PASylated and Fab′ antibodies were similar signifying their similar secondary structure content. In contrast, ΔHm of PEGylated Fab′ was significantly higher than PASylated and Fab′ antibody fragments which may be due to the chemical attachment of PEG moiety to this Fab′ molecule. The initial denaturation temperatures of the three antibodies were approximately equal suggesting that the addition of PEG molecule or fusion of PAS sequences did not change the heat decomposition stability of the Fab′ antibody. The observed descending TGA curves of tested samples showed a weight loss occurrence (Fig. 10b). All three molecules were stable up to 40 °C. In the range of 40–75 °C rapid decomposition and weight loss occurred for Fab′ and the PASylated proteins while degradation temperature of the PEGylated Fab′ slightly shifted to a higher region (80.89 °C). Note that all three tested samples had thermal homogeneity due to the presence of only one peak without any shoulders (Fig. 10) (Table 1).

**Table 1:** Thermal stability characterization of PAS-Fab′, PEG-Fab′ and Fab′ antibodies.

Samples	Denaturation temperature (°C)	ΔT1/2 (°C)	Enthalpy of denaturation (ΔH_m_) (kJ)	Initial decomposition temperature (IDT) (°C)
Fab′	76.11 ± 0.18	16.6 ± 0.15	2.17 ± 0.05	40.0 ± 0.62
PEG-Fab′	80.89 ± 0.26	26.7 ± 0.20	3.05 ± 0.06	40.0 ± 0.85
PAS-Fab′	75.86 ± 0.32	16.6 ± 0.22	2.09 ± 0.07	40.0 ± 0.35

Data have been represented as mean ± SD (three replicate). ΔT1/2 is the temperature when 50% of tested protein has decomposed. IDT is the point where the examined protein started disintegrating and is the measure of thermal stability of that material.

### Pharmacokinetic studies

**Figure 11:**
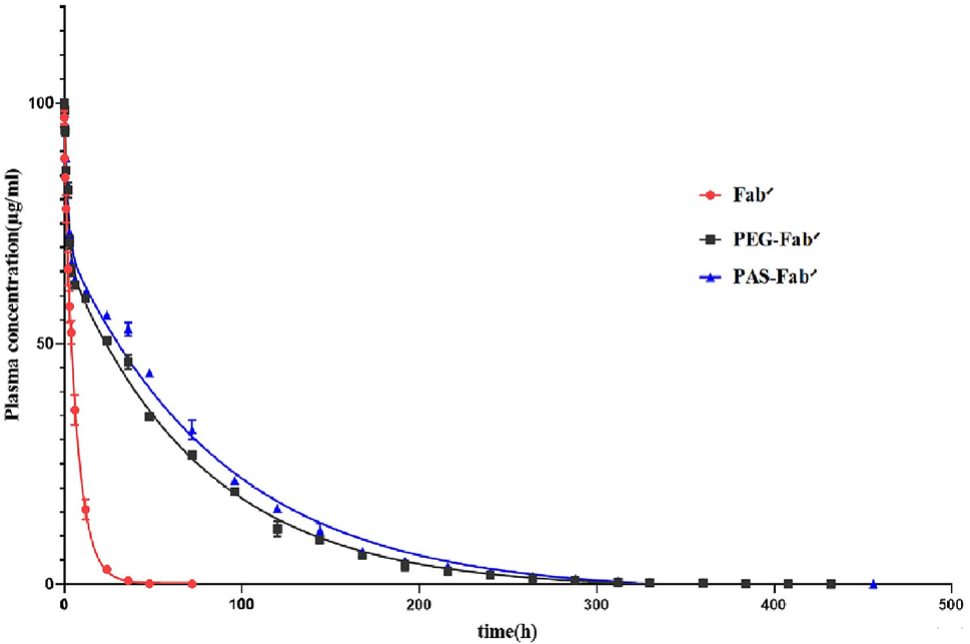
Murine pharmacokinetic profiles of PAS-Fab′, PEG-Fab′, and Fab′ antibodies.

Female BALB/c mice were treated with defined similar concentrations of the three tested proteins to study in vivo pharmacokinetic parameters summarized in Table 2. Terminal plasma half-life (t_1/2_) of Fab′ was 4.622 h revealing quick clearance from blood circulation, while the half-life of the PASylated and PEGylated Fab′ antibodies was 57.28 h and 60.67 h, respectively. The calculated half-lives of PEGylated and PASylated antibodies were very close (12.39 and 12.99 fold longer than the half-life of Fab′ antibody) suggesting that PASylation had a strong effect on improving the terminal half-life of administered Fab′ protein which may be due to the increased hydrodynamic volume of the PASylated protein. The area under the protein concentration–time curve (AUC) indicated that the elimination rate constant and clearance of PASylated and PEGylated Fab′ molecules were very close and significantly better than the Fab′ antibody (Fig. 11) (Table 2).

**Table 2:** In vivo pharmacokinetic parameters of tested PAS-Fab′, PEG-Fab′ and Fab′ antibodies using a log-linear trapezoidal method.

Samples	t_1/2_ (h)	[AUC] 0–∞ (µg h/ml)	Clearance (ml/h kg)	Elimination rate constant (1/h)	PK factor
Fab′	4.622	656.4	7.617	0.602	1
PAS-Fab′	60.67	5730	0.8724	0.03	12.99
PEG-Fab′	57.28	4972	1.005	0.057	12.39

## Discussion

Cimzia is the only anti-TNF-α antibody which is especially administered for patients suffering from Crohn’s disease. Note that this antibody is produced in *E. coli* expression host cells representing lack of the Fc fragment and post-translational modifications such as glycosylation. Cimzia is structurally a Fab′ molecule chemically conjugated to a 40 kDa PEG molecule to increase the half-life of the protein moiety. PEGylation process is a good method to increase half-life but it suffers some disadvantage including laborious, expensive, and time-consuming procedure. Meanwhile, the PEG molecule may induce immunogenicity in the human body or accumulate in renal cells causing vacuolation^22–24^.

To overcome these setbacks, other approaches have been investigated for increasing the protein half-life within the body. Among these methods, PASylation is a newly developed method that lacks the disadvantages of PEGylation^18^. In the present study for the first time, the PASylated form of certolizumab was developed and its biological, physicochemical, and pharmacokinetic properties were assessed and compared with those of the Fab′ as well as PEGylated molecules. To design the expression cassette, PAS sequences including 200 amino acids were added genetically to the C-terminal of both heavy and light chains (400 amino acids in total) given the estimated similar molecular weights of PEGylated antibody molecules. These sequences were attached to the C-terminal of light and heavy chains due to the reactivity of N-terminal ends with TNF-α molecule. The cassette was designed for periplasmic expression of functionally active antibodies through disulfide bond formation between heavy and light chains. Due to the limited periplasmic space, the expression level of PASylated Fab′ was not very significant within this space, while the amount of secreted PASylated Fab′ within the culture medium was approximately 50 times higher than that of the periplasmic space, which may be explained by the increased size of the expressed protein. This finding was supported by the previous studies indicating the effect of prolonged cultivation conditions and high cell density of the culture in leakage of *E. coli* proteins into the culture medium and can be an interesting economical issue in industrial production of such pharmaceutics in *E. coli* in order to enhance the yield of active protein extraction and purification^25–27^.

An exact molecular weight of 82,312.2 Da was measured for PASylated Fab′ using mass spectroscopy, while its electrophoretic mobility was around 200 kDa on SDS-PAGE. This discrepancy has been reported by previous studies indicating the role of hydrophilic amino acids such as alanine, proline, and serine in decreasing SDS-binding properties and mobility of PASylated protein on SDS-PAGE^18,19,25^.

The hydrodynamic volume of a protein is an important parameter affecting the protein renal infiltration and its half-life. Size exclusion chromatography (SEC) represented increased hydrodynamic volume of PASylated Fab′ (~ 416 kDa) which was comparable to the PEGylated form (423 kDa) and much higher than Fab′ (~ 30 kDa) molecule. These results indicated the similar effect of PEGylation and PASylation on increased hydrodynamic volume of certolizumab protein moiety. Mass spectroscopy and SEC revealed a single peak in the purified protein solution indicating the monodispersity of PASylated Fab′, which has not been reported in PEGylated purified proteins^19^. The significantly increased hydrodynamic volume of the PASylated Fab′ molecule is assumed to be related to the random coil structures of PAS motifs observed through CD. The results of CD showed no significant impact on the secondary structure of the molecule. Similar results were reported by the previous studies conducted on other PASylated proteins^18,19^. In order to assess the hydrophobicity of PAsylated protein, RP-HPLC was performed where a single peak of PASylated Fab′ was eluted earlier than the Fab′ control molecule. This result demonstrated that the addition of hydrophilic amino acid residues (proline, alanine, and serine) leads to a decreased hydrophobicity of the molecule^18,28^.

Thermal stability and thermodynamic properties of the expressed PASylated protein were measured through DSC and TGA techniques. The observed quite similar thermal stability pattern and calculated energy of the PASylated Fab′ in comparison to the Fab′ molecule indicated similar internal bonds within these two molecules representing unchanged secondary structure of the PASylated Fab′. Denaturation and initial decomposition temperatures of PASylated and Fab′ molecules were similar, confirming the protein thermal stability in the case of PASylation approach. On the other hand, the increased thermal stability of PEGylated protein may be due to the chemical nature of the PEG moiety and its strong conjugation to the antibody fragment^20,21,29^.

The binding assay by ELISA revealed that the affinity of PASylated Fab' towards TNF-α is 9.46-fold higher than PEGylated form which is in line with the calculated EC50 values. This result reconfirmed the shielding effect of PEG molecules in reducing the binding capacity of the protein to its target molecule (TNF-α in this case) while PASylation did not affect the biological activity of the Fab′ molecule^18,30^.

The calculated affinity constant values of three tested antibodies indicated that the affinity of PASylated form towards TNF-α was similar to the Fab′ molecule and higher than PEGylated Fab′ (3.8-fold). This finding not only confirmed the ELISA results but also proved that PASylation did not have any considerable impact on the affinity of the Fab′ molecule in comparison to the PEGylation method. This finding was in accordance with the previous study reporting higher affinities for PASylated protein in comparison to the corresponding PEGylated form^29^.

Neutralization activity of PASylated Fab′ was similar to that of Fab′ molecule and 2.29-fold stronger than the PEGylated molecule which can be explained by higher binding affinity of PASylated anti-TNF-α to the soluble TNF-α and its inhibition in binding to its receptors on L929 cell line. As noted in ELISA, this result reconfirmed the shielding effect of PEG molecules in decreasing TNF-α binding capacity of the molecule.

In vivo animal studies showed that the pharmacokinetic properties of PASylated Fab′ have been improved compared to the Fab′ molecule (t_1/2_ and clearance remarkably increased by 13.12 and 8.60 times, respectively). This improvement can be explained by the increased hydrodynamic volume of PASylated Fab′ due to the random coil structure of the PAS motif and thus decreased renal filtration of this protein. The pharmacokinetic profile of PEGylated Fab′ was remarkably similar to that of the PASylated form. Previous studies also found longer half-lives for PASylated proteins in comparison to their control molecules^18,31^. Hence, PASylation can be an appropriate alternative approach for increasing the protein half-life.

## Conclusion

According to the literature, few studies have compared the physiological, biological, and pharmacological properties of one protein in both PEGylated and PASylated formats. In this study, however, genetically-fused PAS sequence to the Fab′ molecule resulted in decreased electrophoretic mobility on SDS-PAGE, increased hydrodynamic volume as well as prolonged half-life without affecting the secondary structure of the protein. The expressed PASylated protein had a better biological activity compared to the PEGylated form due to the lack of shielding effect.

Given the drawbacks associated with the PEGylation approach such as immunogenicity, vacuolization within the cells, reduced biological activity, costly and time-consuming multi-step purification processes due to the polydispersity of the PEGylated procedure and high cost of pure PEG, PASylation can be considered as a potential alternative approach in the pharmaceutical industry.

## Methods

### Cloning of PASylated Fab′ fragment

Nucleotide sequences encoding heavy and light chains of the Fab′ antibody fragment as well as PAS sequence type 1 (PAS#1 version: ASPAAPAPASPAAPAPSAPA) were extracted from US20050042219A1 and WO2008155134A1 patents, respectively. The gene cassette containing ribosome binding site (RBS), OmpA signal sequence, heavy chain, the DNA sequence encoding 200 PAS residues and a C-terminal Histidine tag was located between *XbaI* and *NcoI* restriction sites of pET28a expression vector (Novagen, USA). Ribosome binding site (RBS), the gene fragment expressing OmpA signal sequence, and light chain plus 200 PAS residues were inserted between *NcoI* and *HindIII* recognition sites in pET28a vector at downstream of the heavy chain and resulted in the final pET28a Fab′-PAS expression vector (Fig. 1).

### Protein expression

Following protein optimization experiments, pET28a Fab′-PAS expression vector was transformed into BL21 (DE3) *E. coli* strain (Novagen, USA). A single colony was inoculated into 50 ml TB medium supplemented with 50 mg/ml kanamycin,1 g/lit proline, and 6 g/lit glucose, which was incubated at 37 °C overnight in a 150 rpm shaker incubator. The grown bacteria were used to inoculate 2 l TB medium supplemented with the same mentioned solutions and incubated at 30 °C in 200 rpm. The protein expression was induced with 0.5 mM isopropyl β-d-1-thiogalactoside (IPTG) (Sigma, USA) at OD_600nm_ 0.5 and the bacterial pellet was collected 24 h post-induction.

### Extraction of periplasmic PASylated protein

Twenty-four hours after induction, the bacterial pellet was harvested with centrifugation at 5000*g* (15 min). Periplasmic extraction was done through osmotic shock according to the methodology described by Breustedt et al*.*^32^. Briefly, the harvested cells were resuspended in ice-cold periplasmic fractionation buffer (0.5 M sucrose, 1 mM EDTA, 100 mM Tris–HCl, pH8.0 (2 ml/lit OD_600nm_) and incubated in ice for 10 min. In the next step, 15 mM EDTA and 250 μg/ml lysozyme were added from concentrated stock solutions and the cell suspension was incubated for 20 min in ice and centrifuged several times to achieve a clear supernatant containing periplasmic proteins.

### Extraction of PASylated protein from the culture medium

In order to obtain PASylated protein secreted into the medium, the culture medium was concentrated and the buffer was exchanged with KappaSelect binding buffer through Amicon stirred cell (100 kDa NMWL; 200 ml) (Sigma, USA) and Amicon Ultra centrifugal filter units (10 kDa NMWL; 50 ml; Millipore, Billerica, MA).

### Purification of PASylated protein

PASylated Fab′ was purified from extracted periplasmic fraction or the concentrated protein content of the culture medium in a two-step purification procedure using KappaSelect (GE-Healthcare, Canada) followed by Ni–NTA (QIAGEN, Germany) affinity chromatography according to the manufacturer’s instructions. The purified protein was buffer exchanged against phosphate buffer and concentrated using Amicon Ultra centrifugal filter units (10 kDa NMWL).

### SDS-PAGE and Western blotting

The purified proteins were analyzed through 8% SDS-PAGE and western blotting using a prestained high molecular weight protein ladder (Fermentas, Lithuania). The protein concentration was measured using Bradford method with bovine serum albumin (BSA) as the standard protein. For western blotting, proteins were transferred on the nitrocellulose membrane in a wet blotting system (Denagen Tajhiz, Iran). The membrane was blocked with 2% (w/v) skimmed milk (Sigma, USA) in PBS buffer (137 mM NaCl, 3 mM KCl, 8 mM Na_2_HPO_4_, 1 mM KH_2_PO_4_, pH7.4) at 4 °C for 16 h (overnight). The membrane was washed four times with PBS buffer containing 0.05% Tween-20 (PBST) and incubated with anti-Histidine HRP conjugated antibody (1:2000 dilution) (Sigma, USA) at room temperature for 1 h. The membrane was washed five times with PBST buffer and one more time with PBS buffer alone. At the end, the membrane was stained with 3,3′-diaminobenzidine (DAB) (Sigma, USA).

### Secondary structural analysis of PASylated Fab′ molecule

In order to achieve more information about the secondary structure of the newly expressed PASylated Fab′ in comparison to the control molecule (Fab′ fragment), J-810 spectropolarimeter (JASCO Corporation, Japan) was used. The spectra were measured in a wavelength region below 260 nm (195–250 nm) at room temperature (measurement condition: spectral bandwidth 1 nm, scan speed 200 nm/min, response 1 s, using 0.5 mg/ml of each protein in an aqueous solution). The spectra were examined and the molar ellipticity QM was calculated according to the Eq. (1):

1 $${\text{QM}} = \frac{1}{4}\frac{{{\text{Qobs}}}}{{ \left( {{\text{c}}*{\text{d}}} \right)}}$$

where Qobs is the measured ellipticity, c represents the protein concentration (mol/l), and d denotes the path length of the quartz cuvette (1 cm).

### PASylated Fab′ TNF-α binding assay

The binding activity of the expressed PASylated Fab′ and its corresponding control proteins including Certolizumab pegol (UCB, Belgium) as well as the Fab′ molecule was determined toward TNF-α by ELISA. A total of 200 ng/ml TNF-α in PBS was coated in 96-well flat-bottomed ELISA plates and incubated overnight at 4 °C. After blocking the wells with 1% (w/v) BSA in PBS for 2 h at room temperature, 1:2 serial dilutions of PASylated, PEGylated and Fab′ molecules were added to the wells (initial concentration of 300 µg/ml) and incubated for 1 h at room temperature (in triplicate). After the washing step, anti-Kappa HRP conjugated antibody (Sigma, 1:2000 dilution) was added to each well and incubated for 1 h at room temperature. The absorbance was read by a microplate reader (BioTeK, USA) at 450 nm using 3,3′,5,5′-tetramethylbenzidine (TMB) (PishtazTeb, Iran) as the substrate.

### Affinity measurement

The affinity of the recombinant expressed PASylated Fab′ molecule in comparison to the PEGylated form was determined by the method developed by Beatty et al*.*^33^. In brief, 96-well plate was coated with 400 and 800 ng/ml TNF-α at 4 °C overnight. After the blocking step, similar to the mentioned ELISA protocol, serial dilutions of antibodies were added to the wells and incubated for 2 h. According to the obtained optical densities, the affinity constant (*K*_aff_) of each antibody was calculated using the Eqs. (2) and (3)

2 $$\left[ {{{{\text{Ag}}} \mathord{\left/ {\vphantom {{{\text{Ag}}} {{\text{Ag}}^{\prime } }}} \right. \kern-\nulldelimiterspace} {{\text{Ag}}^{\prime } }} = {\text{n}}} \right]$$

3 $${\text{Kaff}} = \frac{{{\text{n}} - 1}}{{2\left( {{\text{n}}\left[ {{\text{Ab}}^{\prime } } \right] - \left[ {{\text{Ab}}} \right]} \right)}}$$

where Ag and Ag′ were set as 800 and 400 ng/ml of TNF-α and Ab and Ab′ values represented antibody EC50 concentrations for the above mentioned antigen concentrations.

### Hydrophobicity analysis of PASylated Fab′

To determine the hydrophobicity of PASylated Fab′ molecule, RP-HPLC column (Hypersil Gold C4; Thermo Fisher Scientific, USA) was used. After equilibration of the C4 column with the buffer containing 2% acetonitrile (ACN) (v/v) and 0.065% trifluoroacetic acid (TFA) (v/v) buffer, approximately 100 µg PASylated, as well as Fab′ proteins were injected. Gradient elution was performed using elution buffer containing 80% ACN (v/v) and 0.05% TFA (v/v) for 30 volumes of the column at a 2 ml/min flow rate. Protein detection was carried out by UV absorption at 280 nm**.**

### Analytical size exclusion chromatography (SEC)

To measure the hydrodynamic volume of the PASylated Fab′ antibody, size exclusion chromatography (SEC) was performed. One hundred microgram of PEGylated, PASylated and Fab′ antibodies were applied to a G3000 SEC column (Tosoh Bioscience, Japan) using phosphate running buffer (0.04 M Na_2_HPO_4_, 0.05 M NaH_2_PO_4_, 0.15 M NaCl, pH7.2) at a flow rate of 0.5 ml/min and chart speed of 1 cm/min. The standard protein marker included bovine thyroglobulin (670 kDa), bovine c-globulin (158 kDa), chicken ovalbumin (44 kDa), horse myoglobin (17 kDa), and vitamin B12 (1.35 kDa). UV absorbance of protein elution was measured at 280 nm.

### Mass spectrometry

To determine the exact molecular weight and monodispersity composition of PASylated Fab′ antibody in comparison to the Fab′ molecule, mass spectrometric analysis (MALDI-TOF/TOF) was performed. In brief, 100 µl (1 mg/ml) PASylated Fab′ protein was desalted by the C18 Zip-Tip reverse phase chromatography pipette tip (Millipore, USA). The sample was mixed with a matrix solution (1:1 v/v of sinapinic acid in 50% ACN containing 0.1% TFA, spotted, air-dried, and analyzed by MALDI-TOF/TOF mass spectrometer (Applied Biosystems 4800 MALDI TOF/TOF, Nd: YAG 200-Hz laser) operated in reflectro positive mode. Data analysis was performed by Data Explorer software version 4.0 (Applied Biosystems, USA).

### Neutralization of soluble TNF-α

To study the cytotoxicity of TNF-α, MTT assay was performed on L929 mouse fibroblast cell line (ATCC CCL-1) cultured in DMEM F12 medium supplemented with 10% heat-inactivated fetal calf serum (FCS), 2 mM glutamine, penicillin (100 units/ml), and streptomycin (0.1 mg/ml) at 37 °C and 5% CO_2_. 2 × 10^4^ cells/well were seeded in 96-well flat-bottom cell culture plates and incubated overnight at 37 °C. Serial dilutions of PASylated, PEGylated, and Fab′ antibodies were prepared and incubated with TNF-α (0.5 ng/ml) and actinomycin D (1 μg/ml) for 2 h at 37 °C. The medium was removed from the wells and 100 μl of the above-mentioned combinations was added to the wells. The plates were incubated overnight at 37 °C. After removal of antigen–antibody complexes, 100 μl of 0.5 mg/ml 3-(4,5 dimethylthiazol-2-yl)-2,5-diphenyl tetrazolium bromide (MTT, Sigma) was added to the wells. The plates were incubated 4 h at 37 °C after which 100 μl SDS (1%) (Sigma, USA) was added to the wells and incubated for further 16 h at 37 °C. The absorbance was measured at 600 nm by a microplate reader spectrophotometer (BioTeK, USA). The wells treated with culture medium alone, actinomycin D alone, and TNF-α alone were tested as controls. Inhibition was calculated using the Eq. (4):

4 $${\text{Inhibition }}\left( \% \right) = \left[ {{{\left( {{\text{V}}_{{\text{Ab/Ag}}} - {\text{V}}_{{{\text{Ag}}}} } \right)} \mathord{\left/ {\vphantom {{\left( {{\text{V}}_{{\text{Ab/Ag}}} - {\text{V}}_{{{\text{Ag}}}} } \right)} {\left( {{\text{V}}_{{\text{N}}} - {\text{V}}_{{{\text{Ag}}}} } \right)}}} \right. \kern-\nulldelimiterspace} {\left( {{\text{V}}_{{\text{N}}} - {\text{V}}_{{{\text{Ag}}}} } \right)}}} \right] \times 100$$

where V_Ab/Ag_ denotes the cellular viability in wells treated with both antibody and TNF-α; V_Ag_ is the cellular viability in wells treated with TNF-α alone, and V_N_ represents the cellular viability in the wells which received neither antibody nor antigen. All tests were done in triplicate.

### Dynamic scanning calorimetry (DSC) and thermogravimetry Assay (TGA)

DSC and TGA as the main analytical techniques were performed using SDT Q600 V.20.9 Build 20 thermal gravimetric and differential thermal analyzer instrument (New Castle, USA) to measure the melting temperature and thermogravimetry of tested proteins, respectively. In brief, PEGylated, PASylated, and Fab′ antibodies were buffer exchanged into phosphate buffer at pH7.4 to a final concentration of 1 mg/ml. The scanning rate was 2 °C/min within the range of 25–150 °C under nitrogen (inert) atmosphere.

### In vivo pharmacokinetic study

This study was confirmed by the Ethics Committee of Pasteur Institute of Iran (EC: IR.PII.REC.1395.42). In vivo pharmacokinetic study was accomplished in accordance with the Declaration of Helsinki ethical principles.

Thirty-two female BALB/c mice (18–20 g) aged 4–6 weeks were purchased from Pasteur Institute of Iran and randomly divided into four groups (n = 8) for pharmacokinetic (PK) studies. The groups were intravenously (*i.v*) injected with 5 mg/kg of PEGylated, PASylated, Fab′ antibodies, and PBS (as negative control) via the tail vein. Blood samples were collected from tail vein after 15 min, 30 min, 1 h, 2 h, 3 h, 6 h, 12 h, 24 h, 36 h, 72 h, 96 h, 120 h, 144 h, 168 h, 192 h, 216 h, 240 h, 264 h, 288 h, 312 h, 336 h, 360 h, 384 h, 408 h, and 432 h post-injection. Serum samples were isolated by centrifugation at 4000 rpm for 10 min at 4 °C and stored at − 20 °C for further analysis. Serum concentration of tested antibody molecules was determined through the home-made ELISA test described in the PASylated Fab′ TNF-α binding assay section. Terminal half-life and pharmacokinetic parameters were calculated using Prism software (v. 8.0).

### Statistical analysis

Statistical analysis was performed by Prism software (v. 8.0). P values below 0.05 were statistically significant. One-way ANOVA test was employed to compare the significant differences between the two experiments.

## Data Availability

All data generated or analyzed during this study have been included in this published article.
